# Layered Oxide Cathodes for Sodium-Ion Batteries: Storage Mechanism, Electrochemistry, and Techno-economics

**DOI:** 10.1021/acs.accounts.2c00690

**Published:** 2023-01-25

**Authors:** Wenhua Zuo, Alessandro Innocenti, Maider Zarrabeitia, Dominic Bresser, Yong Yang, Stefano Passerini

**Affiliations:** † Helmholtz Institute Ulm (HIU), Helmholtzstrassse 11, D-89081 Ulm, Germany; ‡ Karlsruhe Institute of Technology, P.O. Box 3640, 76021 Karlsruhe, Germany; § State Key Laboratory for Physical Chemistry of Solid Surfaces and Department of Chemistry, College of Chemistry and Chemical Engineering, Xiamen University, Siming South Road 422, Xiamen 361005, People’s Republic of China; ∥ Chemistry Department, Sapienza University, Piazzale A. Moro 5, 00185 Rome, Italy

## Abstract

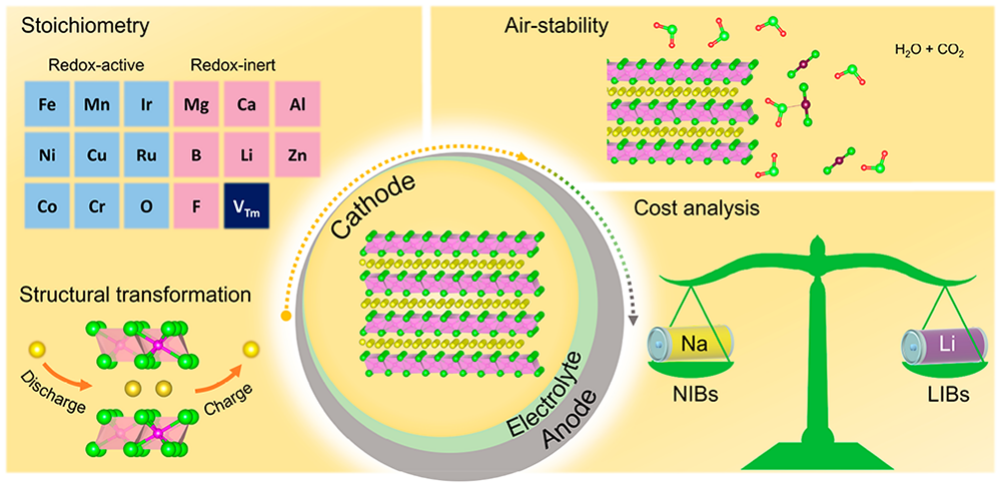

Lithium-ion batteries (LIBs) are ubiquitous in all modern portable electronic devices such as mobile phones and laptops as well as for powering hybrid electric vehicles and other large-scale devices. Sodium-ion batteries (NIBs), which possess a similar cell configuration and working mechanism, have already been proven as ideal alternatives for large-scale energy storage systems. The advantages of NIBs are as follows. First, sodium resources are abundantly distributed in the earth’s crust. Second, high-performance NIB cathode materials can be fabricated by using solely inexpensive and noncritical transition metals such as manganese and iron, which further reduces the cost of the required raw materials. Recently, the unprecedented demand for lithium and other critical minerals has driven the cost of these primary raw materials (which are utilized in LIBs) to a historic high and thus triggered the commercialization of NIBs.

Sodium layered transition metal oxides (Na_*x*_TMO_2_, TM = transition metal/s), such as Mn-based sodium layered oxides, represent an important family of cathode materials with the potential to reduce costs, increase energy density and cycling stability, and improve the safety of NIBs for large-scale energy storage. However, these layered oxides face several key challenges, including irreversible phase transformations during cycling, poor air stability, complex charge-compensation mechanisms, and relatively high cost of the full cell compared to LiFePO_4_-based LIBs. Our work has focused on the techno-economic analysis, the degradation mechanism of Na_*x*_TMO_2_ upon cycling and air exposure, and the development of effective strategies to improve their electrochemical performances and air stability. Correlating structure–performance relationships and establishing general design strategies of Na_*x*_TMO_2_ must be considered for the commercialization of NIBs.

In this Account, we discuss the recent progress in the development of air-stable, electrochemically stable, and cost-effective Na_*x*_TMO_2_. The favorable redox-active cations for Na_*x*_TMO_2_ are emphasized in terms of abundance, cost, supply, and energy density. Different working mechanisms related to Na_*x*_TMO_2_ are summarized, including the electrochemical reversibility, the main structural transformations during the charge and discharge processes, and the charge-compensation mechanisms that accompany the (de)intercalation of Na^+^ ions, followed by discussions to improve the stability toward ambient air and upon cycling. Then the techno-economics are presented, with an emphasis on cathodes with different chemical compositions, cost breakdown of battery packs, and Na deficiency, factors that are critical to the large-scale implementation. Finally, this Account concludes with an overview of the remaining challenges and new opportunities concerning the practical applications of Na_*x*_TMO_2_, with an emphasis on the cost, large-scale fabrication capability, and electrochemical performance.

## Key References

- ZuoW.; QiuJ.; LiuX.; RenF.; LiuH.; HeH.; LuoC.; LiJ.; OrtizG. F.; DuanH.; LiuJ.; WangM. S.; LiY.; FuR.; YangY.The stability of P2-layered sodium transition metal oxides in ambient atmospheres. Nat. Commun.2020, 11, 35443266955810.1038/s41467-020-17290-6PMC7363866.^1^*This article unveils the comprehensive structural/chemical degradation mechanisms of P2-Na*_*x*_*TMO*_*2*_*in different ambient atmospheres by using various microscopic/spectroscopic characterizations and first-principles calculations. A practical evaluating rule associated with redox couples has been proposed for designing air-stable Na*_*x*_*TMO*_2_*cathodes.*
- LiuX.; ZuoW.; ZhengB.; XiangY.; ZhouK.; XiaoZ.; ShanP.; ShiJ.; LiQ.; ZhongG.; FuR.; YangY.P2-Na_0.67_Al_*x*_Mn_1–*x*_O_2_: cost-effective, stable and high-rate sodium electrodes by suppressing phase transitions and enhancing Na^+^ mobility. Angew. Chem., Int. Ed.2019, 58, 18086–1809510.1002/anie.20191169831587462.^2^*In this paper, we introduce Al into the transition metal layers to decrease the number of Mn*^*3+*^*ions and alleviate the phase transformations of Na*_*0.67*_*MnO*_*2*_*. The obtained pure P2-type Na*_*0.67*_*Al*_*x*_*Mn*_*1–x*_*O*_*2*_*(x = 0.05, 0.1, and 0.2) materials show good structural stability and promising performance.*
- ZuoW.; LiuX.; QiuJ.; ZhangD.; XiaoZ.; XieJ.; RenF.; WangJ.; LiY.; OrtizG. F.; WenW.; WuS.; WangM. S.; FuR.; YangY.Engineering Na^+^-layer spacings to stabilize Mn-based layered cathodes for sodium-ion batteries. Nat. Commun.2021, 12, 49033438543510.1038/s41467-021-25074-9PMC8360981.^3^*This paper reports a simple and effective water-mediated strategy to modulate the spacing of Na layers and alleviate undesirable phase transformations of layered oxide cathodes for NIBs. The obtained shale-like material exhibits outstanding rate capability and cycling stability (>3000 cycles)*.
- VaalmaC.; BuchholzD.; WeilM.; PasseriniS.A cost and resource analysis of sodium-ion batteries. Nat. Rev. Mater.2018, 3, 18013.^4^*This work uses the Battery Performance and Cost (BatPaC) model to undertake a cost analysis of the cathodes, anodes, current collector, and complete batteries for NIBs. The detrimental factors for the successful commercialization of NIBs are discussed.*

## 1 Introduction

The world’s leading countries have proposed carbon neutrality by the mid-21st century to relieve the severe effects of worldwide climate change. Achieving this objective requires intensive research efforts in sustainable energy conversion and storage devices. Currently, lithium-ion batteries (LIBs) dominate the energy storage market due to their high energy density and long cycle life. However, the uneven distribution and low abundance of lithium and cobalt raise cost and supply concerns, with the increasing market volume of hybrid electric vehicles (EVs) and stationary storage.^5,6^

Among all the proposed alternative concepts, sodium-ion batteries (NIBs) have the great advantage of being essentially a “drop-in” technology.^7−10^ NIBs and LIBs share the same architecture, similar working mechanisms and components, and identical cell fabrication steps, which implies that NIBs maintain the core of the roll-to-roll system optimized for LIB manufacturing during the last 30 years.

To enable their practical implementation, advanced NIBs with higher energy and power densities, better cycle life and safety, and lower cost are needed. These parameters are correlated to the electrode active materials’ chemical composition, their structure, and reaction mechanisms. After three decades of development, NIBs are at a critical moment of commercialization. Several companies such as HiNa and CATL in China, Faradion in the United Kingdom, Tiamat in France, and NATRON ENERGY in the USA, are close to achieving the commercialization of NIBs, with the aim of employing sodium layered transition metal oxides (Na_*x*_TMO_2_), Prussian white, or vanadium phosphate as cathode materials.^11−14^

Lithium layered transition metal oxides (LiTMO_2_) are the most successful cathode materials for commercial LIBs. Similarly, Na_*x*_TMO_2_ are of particular interest for NIBs due to their high specific capacity, a variety of redox-active elements, and the possibility for the manufacturers to employ established synthesis processes.^15−18^ Other than Na_*x*_TMO_2_, alternative promising sodium-ion cathode materials are Prussian blue/white,^19^ phosphates,^20^ and organic materials.^21^ As already mentioned, materials belonging to these classes have already been selected for the first generation of commercial sodium-ion batteries. Nevertheless, these types of sodium-ion cathodes have the demerits of intrinsic water/potential toxicity, low specific capacity, and/or low capacity retention. However, the “best choice” for the positive active material is still under debate, not least as Na_*x*_TMO_2_-type materials face irreversible phase transitions, electrolyte decomposition, and high reactivity toward moisture. Therefore, a comprehensive evaluation of the chemical composition, electrochemical performance, and cost of Na_*x*_TMO_2_ is fundamental to achieve optimal cathodes for commercial NIBs.

This Account offers an assessment of the currently available Na_*x*_TMO_2_-type materials as candidates for NIBs. First, a fair and accurate comparison of various redox-active elements for Na_*x*_TMO_2_ is given, with a particular focus on availability, energy density, air stability, and structural transformations. Subsequently, a detailed analysis of the cost of Na_*x*_TMO_2_ materials and the resulting cells is provided to illustrate the critical challenges from lab scale toward their practical application. Finally, an outlook and potential future research directions are presented.

## 2 Redox-Active Cations and Structures for Na_*x*_TMO_2_

### 2.1 Abundance, Price, and Energy Density

**Figure 1:**
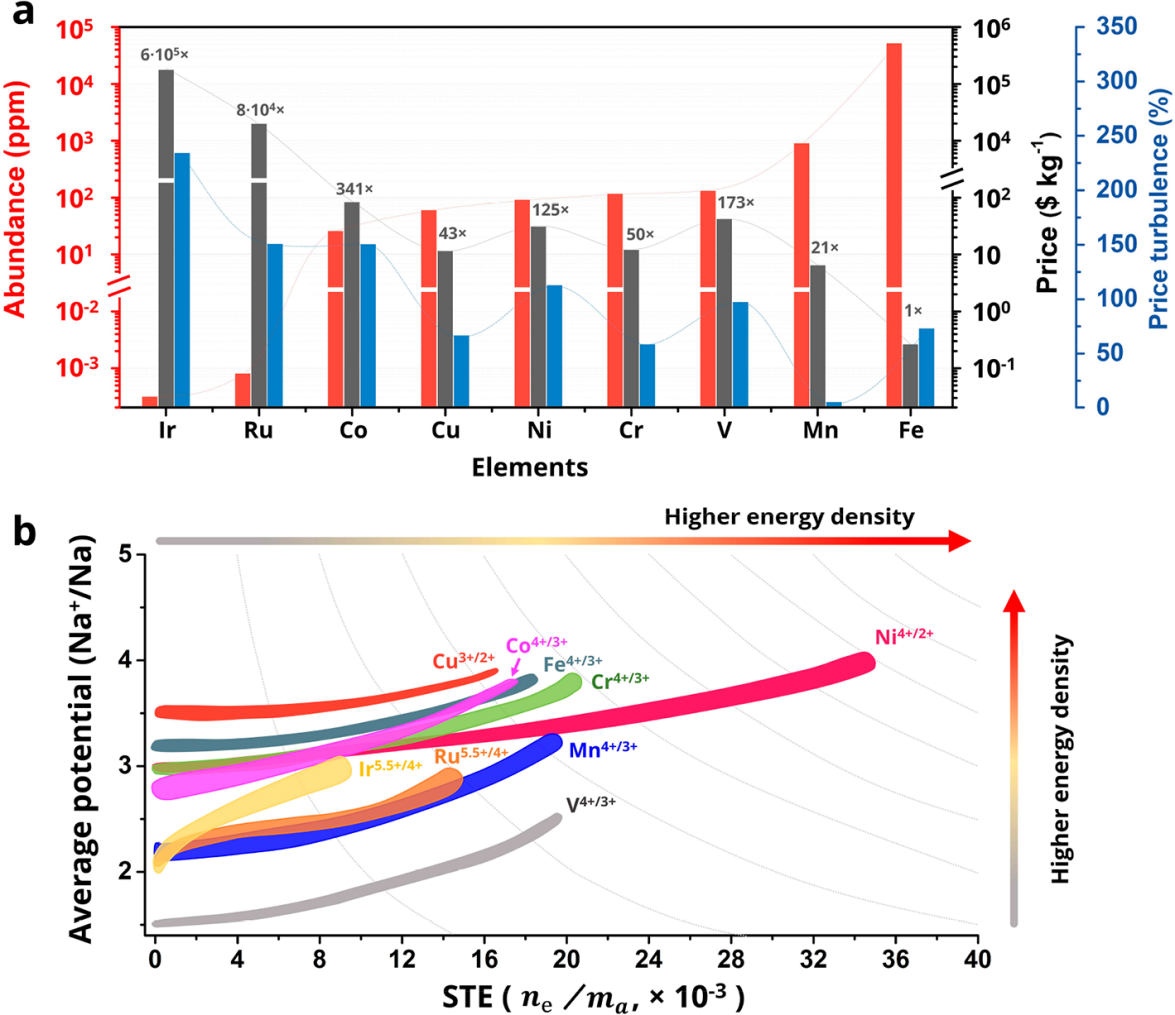
Cationic redox centers of Na_*x*_TMO_2_. (a) Comparison of abundance, the lowest price of ore during January 2020 and April 2022, and the price turbulence from January 2020 to April 2022 of various redox-active elements. (b) Comparison of average working potential, STE, and specific energy density of cationic redox couples.

Figure 1a shows the redox-active cations for Na_*x*_TMO_2_ in the order of their abundance in the earth’s crust, together with their lowest price of ore between January 2020 and April 2022 as well as the price turbulence, calculated as the percentage difference between the lowest and highest prices in this period. Iridium and ruthenium exhibit multielectron reactions during Na (de)intercalation (Figure 1b).^22,23^ However, the high cost and low abundance prevent the implementation of these rare elements in NIBs. Among other TM cations, Fe and Mn have the highest abundance and the lowest price. Mn shows the lowest price turbulence because of its wide distribution in Australia, Asia, America, and Africa. The price and price turbulence are not closely correlated to the abundance in the earth’s crust (Figure 1a) due to the difference in geographical distribution, mining and processing methods, and industrial applications. Although the natural abundances of vanadium and nickel are respectively higher than those of chromium and copper, the price and price turbulence of Cr and Cu are lower than those of Ni and V. Combining the abundance, price, and supply, the favorability of the redox-active cations for Na_*x*_TMO_2_ cathodes follows the order Fe > Mn > Cr > Cu > V > Ni > Co ≫ Ru > Ir. Moreover, since V and Cr are hazardous, their environmental sustainability should be assessed before proposing their commercial utilization.

Considering that the cost per unit energy (energy cost, $ kWh^–1^) determines the competition between NIBs and LIBs instead of the cost per unit weight (mass cost, $ kg^–1^), the contribution of various redox-active cations to the energy density is further evaluated to optimize the chemical compositions of Na_*x*_TMO_2_. A terminology of specific transferred electrons (STE) is defined here as the number of transferred electrons divided by the atomic mass (eq 1):
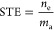
1where *n*_e_ and *m*_a_ are the electrochemically transferred electrons from and the relative atomic mass of the redox-active element, respectively.

Figure 1b shows the correlation of the average working potential with the STE of active redox couples. Cu^3+/2+^ and Ni^4+/2+^ have the highest average working potential, while V^4+/3+^ shows the lowest. After considering STE, the preferred redox couples to achieve a high specific energy density of Na_*x*_TMO_2_ follow the order Ni^4+/2+^ > Cr^4+/3+^ > Fe^4+/3+^, Cu^3+/2+^, Co^4+/3+^ > Mn^4+/3+^ > V^4+/3+^ > Ru^5.5+/4+^ > Ir^5.5+/4+^. Note that the electrochemical reversibility has yet to be considered.

In summary, Fe, Mn, Ni, and Cu are the most favorable elements for constructing a cost-effective and high-energy Na_*x*_TMO_2_ NIB cathode. Mn ions can not only contribute capacity as redox centers but also work as a structural scaffold in the inert Mn^4+^ state.

### 2.2 Air Stability

Besides natural abundances and prices of raw materials, air stability also determines the commercialization of Na_*x*_TMO_2_.^1,24,25^ An air-stable electrode material should maintain its structure, chemical composition, and electrochemical properties after exposure to a specific atmosphere for a given time.^25^ However, because of the lower charge density of Na^+^ and lower redox potential, Na_*x*_TMO_2_ materials display poorer air stability than their lithium counterparts.

**Figure 2:**
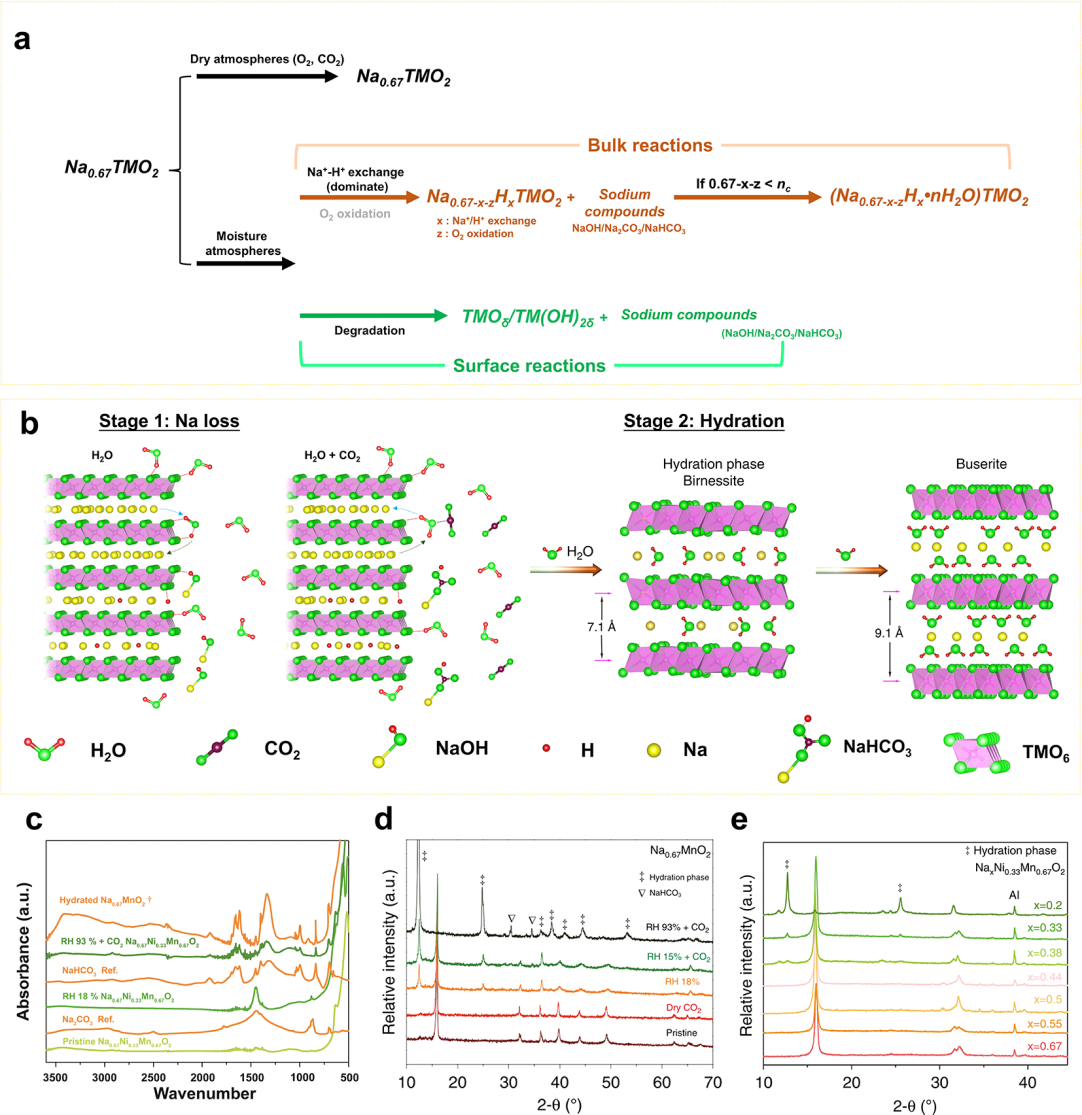
Air stability of Na_*x*_TMO_2_. (a) Summary and (b) schematic illustration of the phenomena occurring upon exposure of Na_*x*_TMO_2_ to moisture. (c) FTIR spectra of moist exposed Na_0.67_MnO_2_ and Na_0.67_Ni_0.33_Mn_0.67_O_2_. (d) XRD patterns of Na_0.67_MnO_2_ exposed to various atmospheres. (e) Relationship between hydration and Na content. From ref (1). CC BY 4.0.

The reactions of Na_*x*_TMO_2_ in moisture are summarized and illustrated in Figure 2a,b. As revealed by our previous works,^1,25^ once Na_*x*_TMO_2_ is exposed to moist air, H_2_O(g) tends to be adsorbed on the particles’ surface and/or react with CO_2_ to form H_2_CO_3_(ad) (Figure 2b, stage 1). Those H_2_O(ad) and H_2_CO_3_(ad) dissociate into H^+^, OH^–^, HCO_3_^–^, and CO_3_^2–^, react with Na^+^ in the surface layer, and produce NaOH, NaHCO_3_, and Na_2_CO_3_ (Figure 2c,d).^1,25^ These chemical reactions accelerate the Na^+^ extraction from Na_*x*_TMO_2_. After the extraction of a large amount of Na^+^, water molecules insert into the Na layers and form hydration phases (Figure 2d). Furthermore, under extreme conditions, severe hydration processes might occur and form buserite phases. Besides these reactions, the surface layer of some Na_0.67_MnO_2_ materials reacts with moisture, forming TM oxides and hydroxides (Figure 2a).^1,25^

Our research suggests that the air stability of Na_*x*_TMO_2_ cathodes is usually determined by four factors: (i) chemical composition and (ii) structure of the material, (iii) temperature, and (iv) atmospheric conditions (such as pressure, relative humidity, and the gas components).^1,25^ The chemical composition and structure determine the working potential and formation energy of the materials, which further influence the feasibility of Na loss in moisture and therefore the air stability.^1^

In general, Na_*x*_TMO_2_ materials with lower working potentials during the first charge process are more vulnerable to Na loss and hydration (Table 1).^1^ For example, Co-rich and Mn-rich Na_*x*_TMO_2_ materials, in which Co and Mn are redox-active in the initial charge process, suffer from Na loss and can be easily hydrated. Nevertheless, they exhibit better stability toward the formation of TM oxides and hydroxides compared to Cu-, Ni-, Fe-, and Cr-rich Na_*x*_TMO_2_.

**Table 1:** Summary of Na_*x*_TMO_2_ Based on Various Redox Couples

	air stability^a^				
redox couple	risk of Na loss	risk of hydration	risk of metal oxide formation	disadvantages	favorability for implementation
Ni^4+/2+^	low	low	high; NiO in Ni-rich samples	severe phase transformations	high
O^*n*–/2–^	low	low	medium	large voltage hysteresis	medium
Cu^3+/2+^	low	low	high; Cu_2_O in Cu-rich samples	restricted specific capacity	high
Fe^4+/3+^	high	medium	high; FeOOH	degrades faster with higher Fe content	high, but with low content
Cr^4+/3+^	high	medium	high	hazardous; severe structural changes at low Na content	medium
Co^4+/3+^	high	medium	low	high cost	low
Mn^4+/3+^	high	high	low	low working potential	high
V^4+/3+^	high	high	unknown	hazardous; low working potential	low
Ir^5.5+/4+^	high	unknown	high	high cost and low abundance	none
Ru^5.5+/4+^	high	unknown	high	high cost and low abundance	none

a Corresponding to the original materials instead of the charged/discharged states.

### 2.3 Structural Transformations

The electrochemical reversibility of Na_*x*_TMO_2_ is determined by the structural stability and electrode–electrolyte interface. When assessing the availability of a cathode, structural stability is usually more critical than electrolyte depletion. For example, we found that the capacity of pristine P2-type Na_0.67_MnO_2_ fades to 41% of the initial discharge capacity after 100 cycles because of structural degradation.^2,26^ The modified Na_0.67_MnO_2_ with good structural reversibility could work for hundreds to thousands of cycles, and the critical factor that determines electrochemical reversibility shifts from structure to electrolyte.^3^

**Figure 3:**
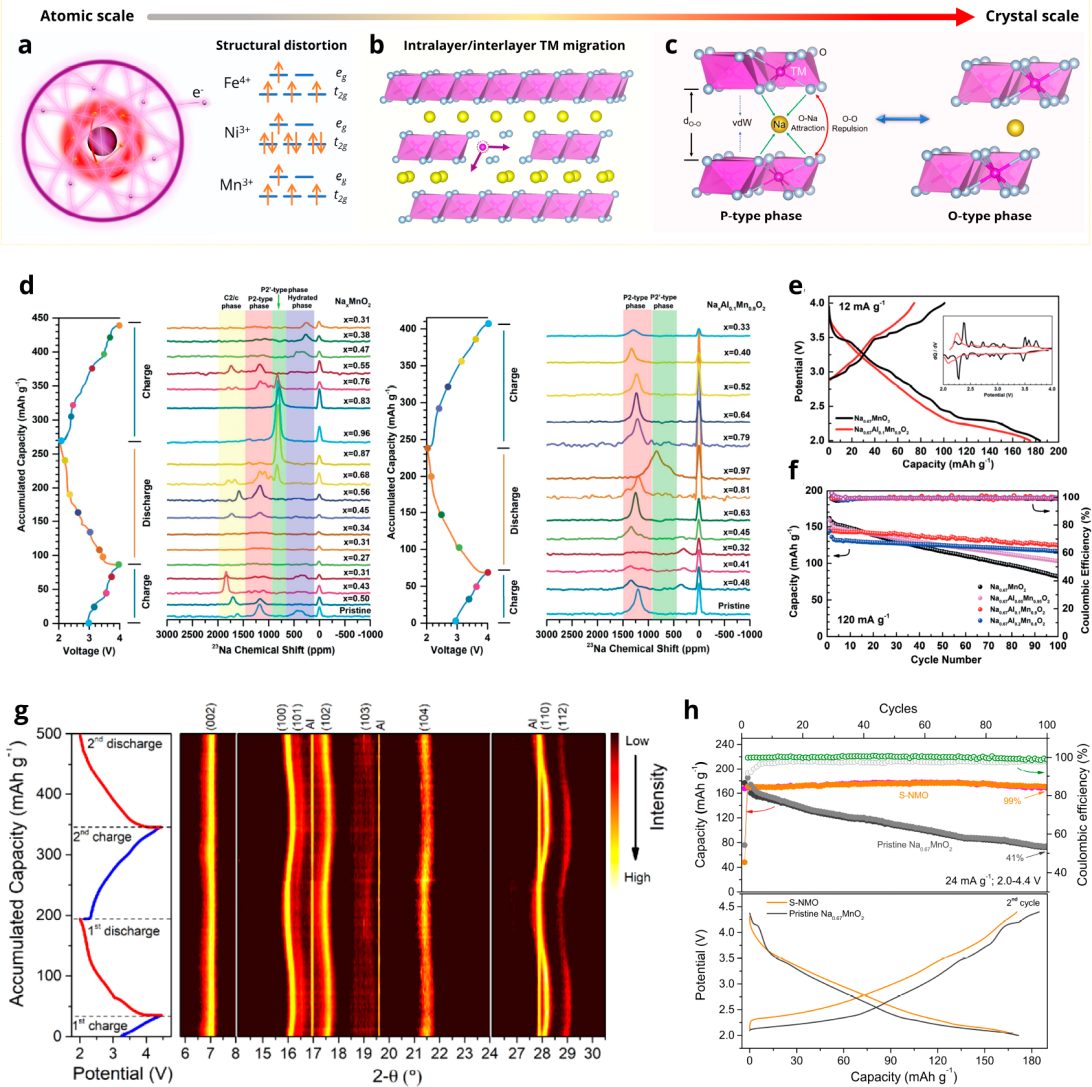
Structural transformations of Na_*x*_TMO_2_. (a–c) Schematic illustrations of the (a) structural distortion, (b) TM migrations, and (c) P-type and O-type phase transitions. (d) Ex situ SS-NMR spectra of Na_*x*_MnO_2_ and Na_*x*_Al_0.1_Mn_0.9_O_2_, (e) initial charge–discharge curves of Na_0.67_MnO_2_ and Na_0.67_Al_0.1_Mn_0.9_O_2_, and (f) cycling performance of Na_0.67_Al_*x*_Mn_1–*x*_O_2_ (*x* = 0, 0.05, 0.1, 0.2). (g) In situ synchrotron XRD patterns and (h) electrochemical performance of S-NMO and Na_0.67_MnO_2_. (a–f) Reproduced with permission from ref (2). Copyright 2019 Wiley-VCH. (g, h) From ref (3). CC BY 4.0.

The phase transformations of Na_*x*_TMO_2_ can be attributed to three main factors, as shown in Figure 3a–c. The first factor is the electronic structural changes of cations and oxygen that lead to anisotropic structural change or distortion (Figure 3a). Typical examples are Jahn–Teller (JT)-active Mn^3+^, Fe^4+^, and Ni^3+^ with octahedral coordination. JT distortion is a primary phase transformation for Mn-rich Na_*x*_TMO_2_ (Figure 3d), which leads to a significant volume change and slow kinetics. A common mitigation approach is to destroy the continuity of Mn ions and transform long-range JT distortion into a local one. As shown in our solid-state nuclear magnetic resonance (SS-NMR) spectra results in Figure 3d, Na_0.67_MnO_2_ suffer from multiple phase transformations. After Al is introduced into the TM layers, the phase transitions are alleviated, thus resulting in much-improved electrochemical performance (Figure 3 e,f).^2^ JT distortions of Fe and Ni redox reactions have been rarely reported because Na_*x*_FeO_2_ and Na_*x*_NiO_2_ compounds suffer from other severer phase transitions and poor air stability,^27^ whereas for the Ni- or Fe-contained Na_*x*_TMO_2_, such as Na_*x*_Ni_0.33_Mn_0.67_O_2_ and Na_*x*_Fe_0.5_Mn_0.5_O_2_, the discontinuous distribution of Ni and Fe thwarted the influence of JT-active ions on the long-range structural changes.^2,28,29^

Cation migration (Figure 3b) is another intrinsic factor that causes structural transformation of Na_*x*_TMO_2_. During charge–discharge processes, the interlayer cation migration transforms layered Na_*x*_CrO_2_,^30^ Na_*x*_VO_2_,^31^ and Na_*x*_FeO_2_^27,32^ into disordered or rock-salt structures when the Na content is lower than ∼0.5 (Table 2), which leads to poor electrochemical reversibility. These cation migrations could be mitigated by adjusting the chemical composition or the upper cutoff potential. TM migration also poses an inherent challenge for Mn-based Na_*x*_TMO_2_. Intralayer and interlayer TM migrations caused by TM vacancies and anionic redox of Mn-based Na_*x*_TMO_2_ result in gradual capacity fade and large voltage hysteresis.

**Table 2:** Summary of Structural Transformations of Various Redox Couples for Na_*x*_TMO_2_

redox couple	main structural transformations	intrinsic electrochemical reversibility
Ni^4+/2+^	P–O phase transition; large volumetric change	excellent for Ni^3+/2+^ redox couple, poor for Ni^4+/3+^
O^*n*–/2–^	TM migration; oxygen loss	highly dependent on chemical composition
Cu^3+/2+^	P–O phase transition	generally good unless working at the high voltage
Fe^4+/3+^	P–O phase transition; TM migration; oxygen loss	dependent on Fe content
Cr^4+/3+^	P–O phase transition; TM migration	good for Cr^3.5+/3+^ redox couple and poor for Cr^4+/3.5+^
Co^4+/3+^	oxygen loss	good for Co^3.5+/3+^ redox couple and poor for Co^4+/3.5+^
Mn^4+/3+^	structural distortion; P–O phase transition	poor for either very low or very high sodium contents while excellent for medium sodium content
V^4+/3+^	TM migration	good for V^3.5+/3+^ redox couple and poor for V^4+/3.5+^

Third, changes in the balance between electrostatic interactions of O–O repulsion, O–Na attraction, and van der Waals (vdW) force lead to phase transitions from P-type to O-type structures (Figure 3b).^3^ The most common structural change in Na_*x*_TMO_2_ are P–O phase transitions, which usually cause large volume changes, fast structural degradation, and capacity fading.^33^ P–O transitions might be mitigated but are unlikely to be phased out by cation substitution.^26,34−36^ Recently, we developed a water-mediated strategy^3^ to modify the spacing of Na^+^ layers of Na_*x*_TMO_2_ and weaken the electrostatic interactions. As shown in Figure 3g, the modified Na_*x*_TMO_2_ (S-NMO) exhibits little structural change except for the breath effect aroused by Mn valence change and shows excellent electrochemical reversibility (Figure 3h).^3^

Oxygen loss is a well-known instability issue that originates from two mechanisms: First, like Li-rich oxides,^37,38^ the intralayer migration of TM ions leads to the formation of O_2_ and bulk holes. Second, at high working potentials, the surface oxygen ions of Na_*x*_TMO_2_ become highly active, react with the electrolyte, and generate O_2_ gas.

Table 2 summarizes the electrochemical reversibility and main structural transformations of different redox couples. Remarkably, the electrochemical and structural properties do not depend solely on the main redox-active elements but also on the presence of other ions and their mutual interactions. Moreover, most of the structural transformations could be mitigated by regulating the chemical composition, introducing redox inert cations, and modulating the Na layer spacing.^3,16,29^

### 2.4 Electrolyte

Carbonate-based solvents with NaFP_6_ and NaClO_4_ as Na salts are the most widely used liquid electrolytes for NIBs.^39,40^ Despite the acceptable electrochemical performance, the continuous growth of the cathode–electrolyte interphase (CEI) in carbonate electrolytes upon cycling impoverishes the electrochemical properties of Na_*x*_TMO_2_, as our group demonstrated previously in different Na_*x*_TMO_2_ chemistries.^3,26,41−43^ For example, the inorganic-rich carbonate and alkyl carbonate CEI is highly soluble in the electrolyte due to their mild Lewis acidity,^41^ which leads to battery degradation.^42,43^

**Figure 4:**
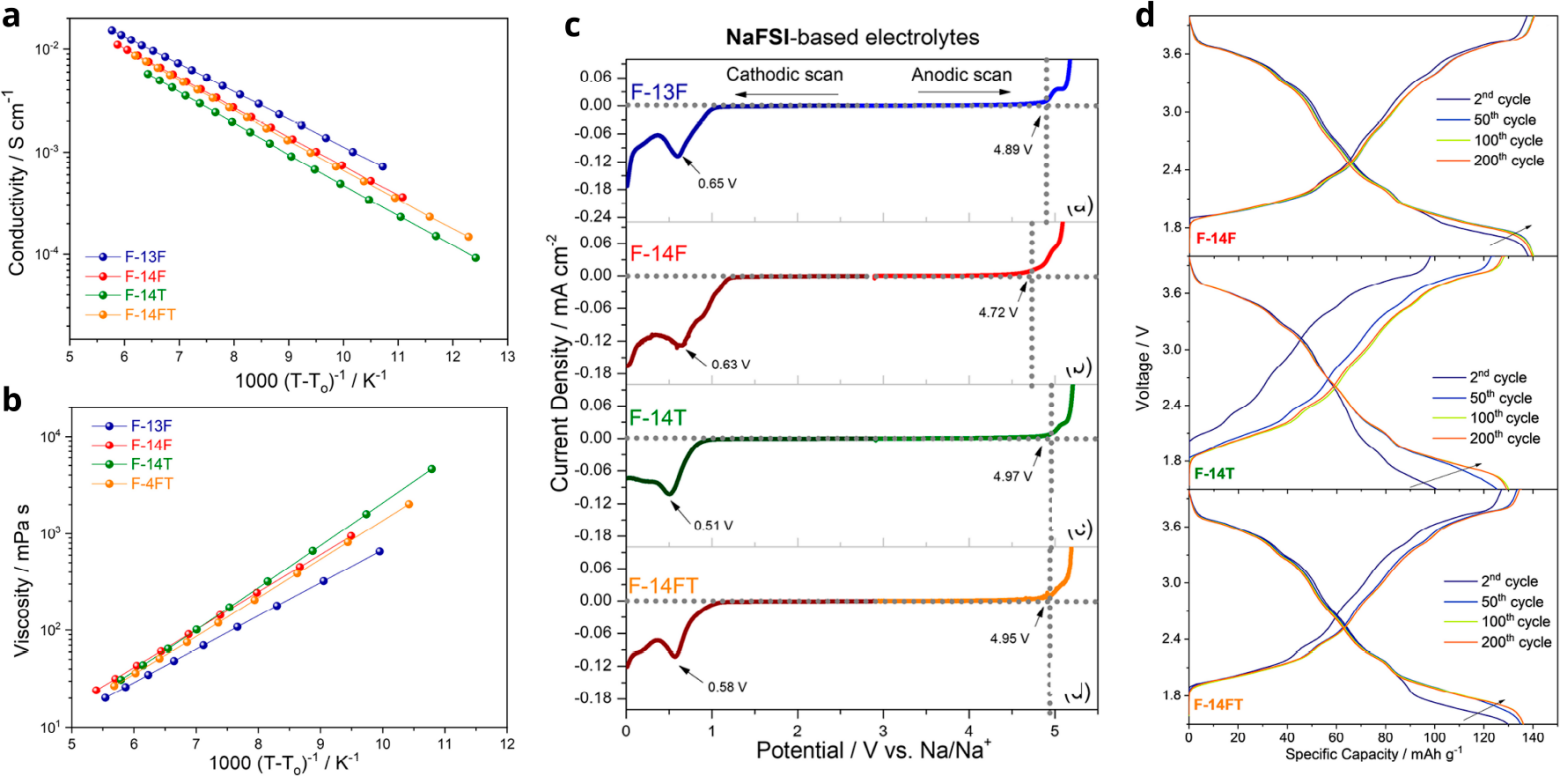
Structural transformations of Na_*x*_TMO_2_. (a) Conductivity and (b) viscosity Volgel–Tammann–Fulcher (VTF) plots for NaFSI-based electrolytes. (c) Electrochemical stability window of NaFSI-based electrolytes. (d) Galvanostatic charge–discharge profiles at selected cycles of Na_0.6_Ni_0.22_Al_0.11_Mn_0.66_O_2_//Na cells within 1.5–4.0 V in NaFSI-based IL electrolytes. F-13F: 10 mol % NaFSI in Pyr_13_FSI. F-14F: 10 mol % NaFSI in Pyr_14_FSI. F-14T: 10 mol % NaFSI in *N*-butyl-*N*-methyl-pyrrolidinium bis(trifluoromethanesulfonyl)imide (Pyr_14_TFSI). F-14FT: 10 mol % NaFSI in 4:5 mol/mol Pyr_14_FSI/Pyr_14_TFSI. Reproduced from ref (45). Copyright 2019 American Chemical Society.

On the contrary, ionic liquid (IL)-based electrolytes have a wider electrochemical stability window, superior thermal stability, and negligible flammability and volatility.^42,44^ Our group tested the physicochemical properties of a variety of fluorinated-IL electrolytes. As shown in Figure 4a,b,^45^ the IL electrolytes sodium bis(fluorosulfonyl)imide (NaFSI) in *N*-methyl-*N*-propylpyrrolidinium bis(fluorosulfonyl)imide (Pyr_13_FSI), denoted as F-13F, and NaFSI in *N*-butyl-*N*-methylpyrrolidinium bis(fluorosulfonyl)imide (Pyr_14_FSI), denoted as F-14F, exhibit a very low tendency to crystallize at room/subambient temperature and a high ionic conductivity above 10^–3^ S cm^–1^. Moreover, as shown in Figure 4b,^45^ F-13F exhibits superior anodic stability of 4.89 V. In contrast, the other studied chemistries show inferior anodic stabilities of 4.72, 4.97, and 4.95 V for F-14F, F-14T, and F-14FT, respectively. Among various ILs, the F-14F (10 mol % NaFSI in Pyr_14_FSI) electrolyte enabled outstanding electrochemical performance in Na_*x*_TMO_2_ cathodes (Figure 4d),^45^ suggesting that IL electrolytes can be a good alternative as electrolytes for NIBs. Indeed, our previous study confirmed that the long-term stability of Na_*x*_TMO_2_ is enhanced by replacing the carbonate-based liquid electrolyte with an F-14F IL electrolyte while maintaining the specific capacity.^42^ The better stability was attributed to a homogeneous and stable CEI. Cost is one of the most critical issues of ILs. Nevertheless, ILs can be easily recycled, and some manufacturing companies, such as Solvionic, are carrying out great efforts to reduce their cost.

## 3 Cost Analysis

Compared to LIBs, the absence of cost-sensitive elements like Li and Co and the reduction of Ni content in Na_*x*_TMO_2_ cathodes should lead to a much lower cost for NIBs.^6^ However, the previous cost analysis made by our group on both materials and complete batteries opposed this assumption.^4^ Recently, the price of the main raw materials for LIBs has risen drastically (Figure 1a) due to the pandemic crisis and geopolitical instabilities, while the main raw materials for NIBs have experienced a smaller price increment. For instance, the price of Li_2_CO_3_ passed from 7.20 $ kg^–1^ in January 2020 to 78.00 $ kg^–1^ in April 2022, whereas Na_2_CO_3_ cost 0.25 $ kg^–1^ and 0.40 $ kg^–1^ on the same dates, respectively. Therefore, the costs of current LIBs and NIBs would differ substantially from those of 2 years ago.

**Figure 5:**
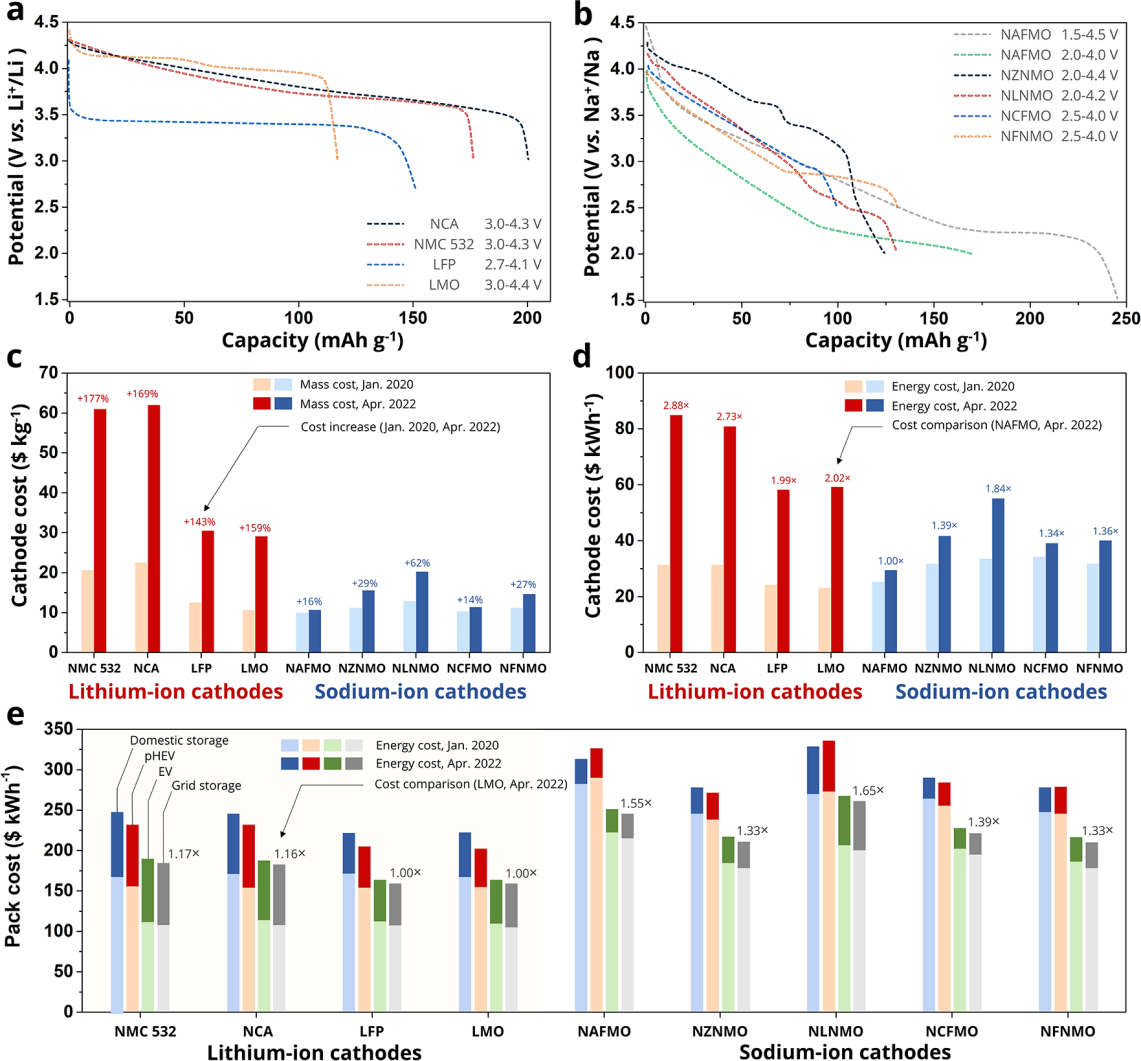
Cost analysis of Na_*x*_TMO_2_ cathodes and battery packs including them. (a, b) Initial discharge curves of (a) Li cathodes and (b) Na cathodes from the references. (c, d) Comparison of (c) cost per unit mass and (d) cost per unit energy between lithium-ion and sodium-ion cathodes. (e) Costs of battery packs based on various lithium- and sodium-cathode materials for different types of applications. Data were taken from refs (34), (35), (47), (49), and (50).

As discussed in the above section, the most favorable TM elements for commercial Na_*x*_TMO_2_ are Fe, Mn, Ni, and Cu. Here, we evaluated the cost of five promising Na_*x*_TMO_2_ cathodes and corresponding batteries,^46^ i.e., P2-type Na_0.67_[Al_0.1_Fe_0.05_Mn_0.85_]O_2_ (NAFMO),^47^ P2-type Na_0.66_[Ni_0.26_Zn_0.06_Mn_0.67_]O_2_ (NZNMO),^34,48^ O3-type Na[Li_0.10_Ni_0.35_Mn_0.55_]O_2_ (NLNMO),^35^ O3-type Na_0.9_[Cu_0.22_Fe_0.30_Mn_0.48_]O_2_ (NCFMO),^49^ and O3-type Na[Fe_0.40_Ni_0.30_Mn_0.30_]O_2_ (NFNMO),^50^ compared to LIBs based on Li[Ni_0.5_Mn_0.3_Co_0.2_]O_2_ (NMC 532), Li[Ni_0.8_Co_0.15_Al_0.05_]O_2_ (NCA), LiMn_2_O_4_ (LMO), and LiFePO_4_ (LFP) cathodes coupled with graphite. The initial discharge curves of all cathodes are presented in Figure 5a,b.

### 3.1 Cost Analysis of Cathodes and Battery Packs

Figure 5c,d and Table S1 show the mass/energy costs of the evaluated cathodes. In 2020, the mass costs of the evaluated Na cathodes follow the order NLNMO (12.48 $ kg^–1^) > NZNMO > NFNMO > NCFMO > NAFMO (10.22 $ kg^–1^), which are comparable to those of LFP (12.46 $ kg^–1^) and LMO (11.16 $ kg^–1^). Two years after, the cost difference between these cathode materials increased substantially. The costs of Li cathodes increased by at least 143%, while those of NAFMO and NCFMO only increased by 16% and 14%, respectively. The mass cost increment of NLNMO is 62%, which is the highest among the Na cathodes due to the presence of Li. As a result, the mass costs of Li cathodes have become significantly higher than those of Na cathodes. Note that the content of Co of the considered Li cathodes is very low; otherwise, the difference in cost would be greater.

Similar trends are observed in energy cost. In 2020, NAFMO and NLNMO showed the lowest and highest energy costs of 25.34 and 33.76 $ kWh^–1^ among Na cathodes, respectively, which are higher than those of LFP (24.21 $ kWh^–1^) and LMO (23.43 $ kWh^–1^). Two years later, the energy costs of the five Na cathodes follow the order NLNMO (54.56 $ kWh^–1^) > NZNMO (41.11 $ kWh^–1^) > NFNMO (40.42 $ kWh^–1^) > NCFMO (39.77 $ kWh^–1^) > NAFMO (29.67 $ kWh^–1^), which are lower than those of LFP (58.91 $ kWh^–1^) and LMO (59.94 $ kWh^–1^), demonstrating that the current Na_*x*_TMO_2_ cathodes have an overwhelming cost advantage over Li cathodes.

Figure 5e presents the costs of 36 different battery packs based on the price of raw materials in January 2020 and April 2022. Four different battery sizes are considered here (Table S2), i.e., a domestic battery pack of 7 kW and 11.5 kWh, a plug-in hybrid EV (pHEV) pack of 110 kW and 15 kWh, a high-end EV pack of 150 kW and 100 kWh, and a grid-storage pack of 250 kW and 500 kWh. LFP-/LMO-based packs show the lowest costs, followed by NMC-/NCA-based batteries. With increased pack energy, the energy cost of LIBs decreases due to economy of scale effects and the lower impact of the hardware costs (casings, battery management system, and cooling system). The energy costs of all NIBs are higher than those of LIBs. NZNMO-/NFNMO-based battery packs for grid storage show the lowest energy cost among the NIB packs, but it is still 33% higher than that of LFP-/LMO-based grid storage packs. NAFMO has the lowest mass and energy cost among the considered Na cathodes. However, the pack costs of NAFMO-based NIBs are much higher than those of NZNMO, NCFMO, and NFNMO because of a lower working potential and increased positive electrode porosity due to the presence of the sacrificial salt. NLNMO has the highest cost among the Na cathodes, and NLNMO-based batteries show the highest pack costs, demonstrating that high ratio of Li, such as 10%, should be avoided in cost-effective Na cathodes.

### 3.2 Cost Differences between NIBs and LIBs

The current raw material costs of Na cathodes are substantially lower than those of Li cathodes. However, the pack costs of NIBs are still higher than those of LIBs (Figure 5 and Tables S3 and S4). To clarify the cost gap between NIBs and LIBs, cost breakdowns of grid-storage packs are analyzed more in detail.

**Figure 6:**
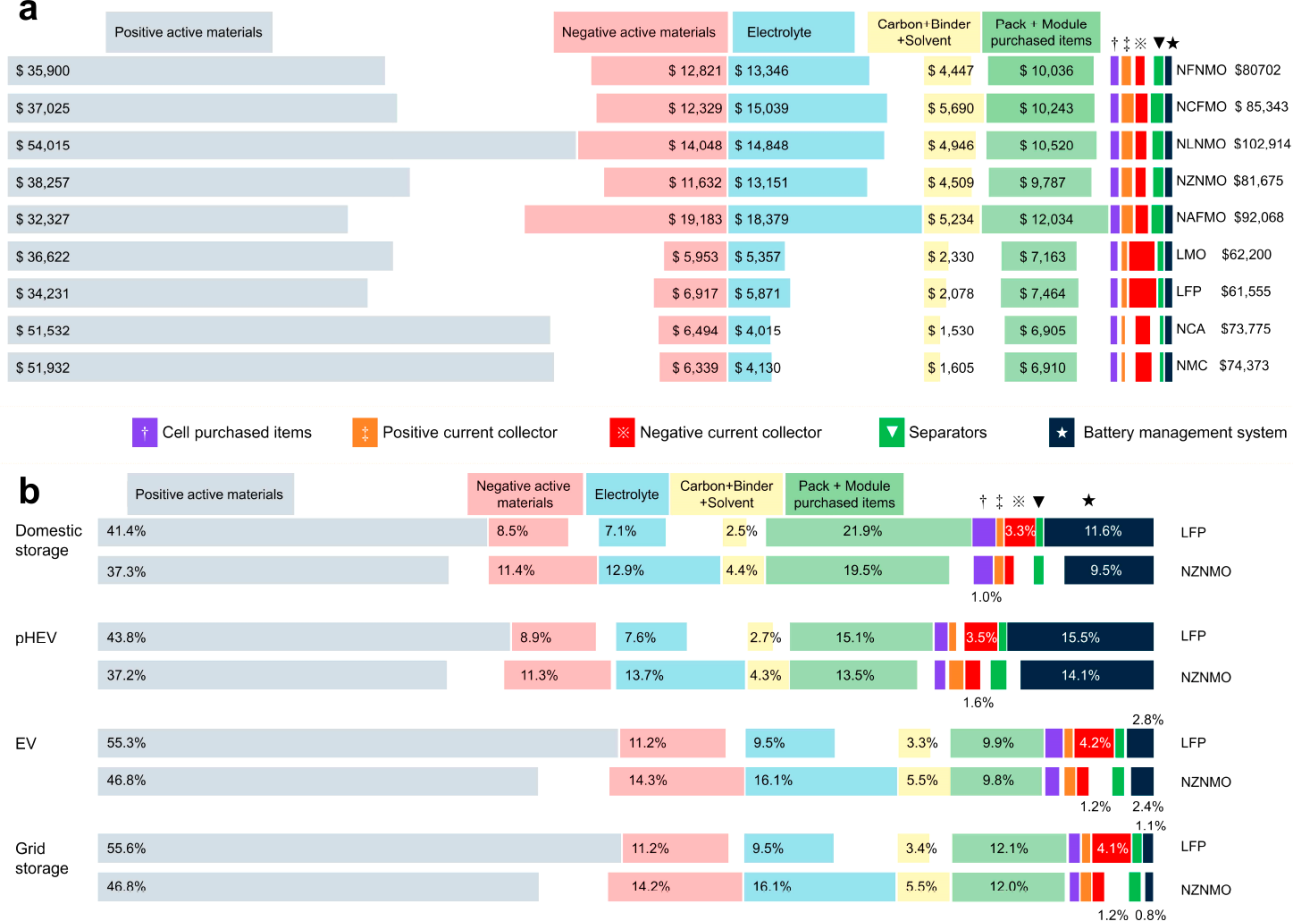
Cost breakdown of LIBs and NIBs. (a) Cost breakdown of various NIBs and LIBs for grid storage. (b) Cost breakdown of LiFePO_4_ (LFP)-based and Na_0.67_Zn_0.06_Ni_0.26_Mn_0.67_O_2_ (NZNMO)-based battery packs in April 2022.

As presented in Figure 6a, the positive active materials, negative active materials, electrolyte, pack and module purchased items, and electrode-preparation compounds (carbon + binder + solvent) occupy over 90% of the pack cost. Except for the NLNMO-based battery, the cathode costs of the evaluated NIBs are comparable to or even lower than those of LMO-/LFP-based batteries. However, the negative active material costs much more in NIBs than in LIBs because of the higher average working potential of hard carbon than graphite and the lower working potential of Na cathodes. For example, the hard carbon costs $19,183 in NAFMO-based NIBs, which is 3 times the cost of graphite in LFP-based LIBs. Besides, the lower energy densities of both the cathode and anode for NIBs require higher amounts of electrolyte, electrode-preparation compounds, and the pack + module purchased items, leading to unfavorable pack cost of NIBs.

With the higher amount of positive active material, the cost of the NIB packs might be significantly reduced by transitioning from poly(vinylidene difluoride) (PVdF) dissolved in *N*-methyl-2-pyrrolidone (NMP) to water-soluble binders due to the cost advantages of water over NMP (70–200 times), water-soluble and F-free polymers over PVdF (2–5 times), and the lower energy needed for the electrode drying process.^51,52^ The challenge of incorporating water-soluble binders lies in the high reactivity of Na_*x*_TMO_2_ toward moisture.^1,25^ However, our group recently reported superior cycling stability and (de)sodiation kinetics for P2-Na_*x*_TMO_2_ materials due to the removal of carbonate surface species during the electrode processing.^3^ Furthermore, better surface coverage of the active material particles by the binder, suppressing electrolyte decomposition, and enhanced mechanical integrity of such electrodes could be achieved via the interaction with the hydroxyl/carboxyl groups in such water-soluble polymers as well as the reduced swelling with the electrolyte.^53,54^

LFP-based LIBs and NZNMO-based NIBs are further selected to compare the cost proportions of pack components (Figure 6b). With the increment in energy of battery packs, the cost proportions of the electrode active materials, electrolyte, and electrode-preparation compounds increase, while the ones of purchased items and hardware decrease. The positive active material occupies a smaller cost proportion in NIBs than in LIBs. On the contrary, the balance of negative active material, electrolyte, and electrode-preparation components is higher than that of LIBs. Moreover, more current collectors and separators are required for NIBs, leading to a higher cost for positive current collectors and separators. The cost proportion of the negative current collector in NIB packs is 2–3% lower than in LIB packs, as the expensive Cu could be replaced by Al.

In summary, Na_*x*_TMO_2_-based NIBs have cost advantages for the positive active material and negative current collector, which are outbalanced by the higher costs of other battery components.

### 3.3 Sodium Deficiency in Na_*x*_TMO_2_

The stoichiometry of sodium in Na_*x*_TMO_2_ is in the range of 0.6 ≤ *x* ≤ 1.0,^19^ and after-synthesis treatments or air exposure can bring this initial sodium content to an even lower value.^1,3^ In commercial setups, the cathode is the only Na source for the whole battery, and low Na content in Na_*x*_TMO_2_ might lead to severe Na deficiency in full cells. Moreover, the low initial Coulombic efficiency (ICE) of hard carbon further decreases the quantity of available sodium, making the issue of Na deficiency more complicated. In a work of our group on bio-based hard carbons, the ICE of a peanut-shell-derived hard carbon was around 70%, and the best commercial hard carbons achieve slightly more than 80%, while the graphite of LIB anodes can easily reach 90%.^55,56^

**Figure 7:**
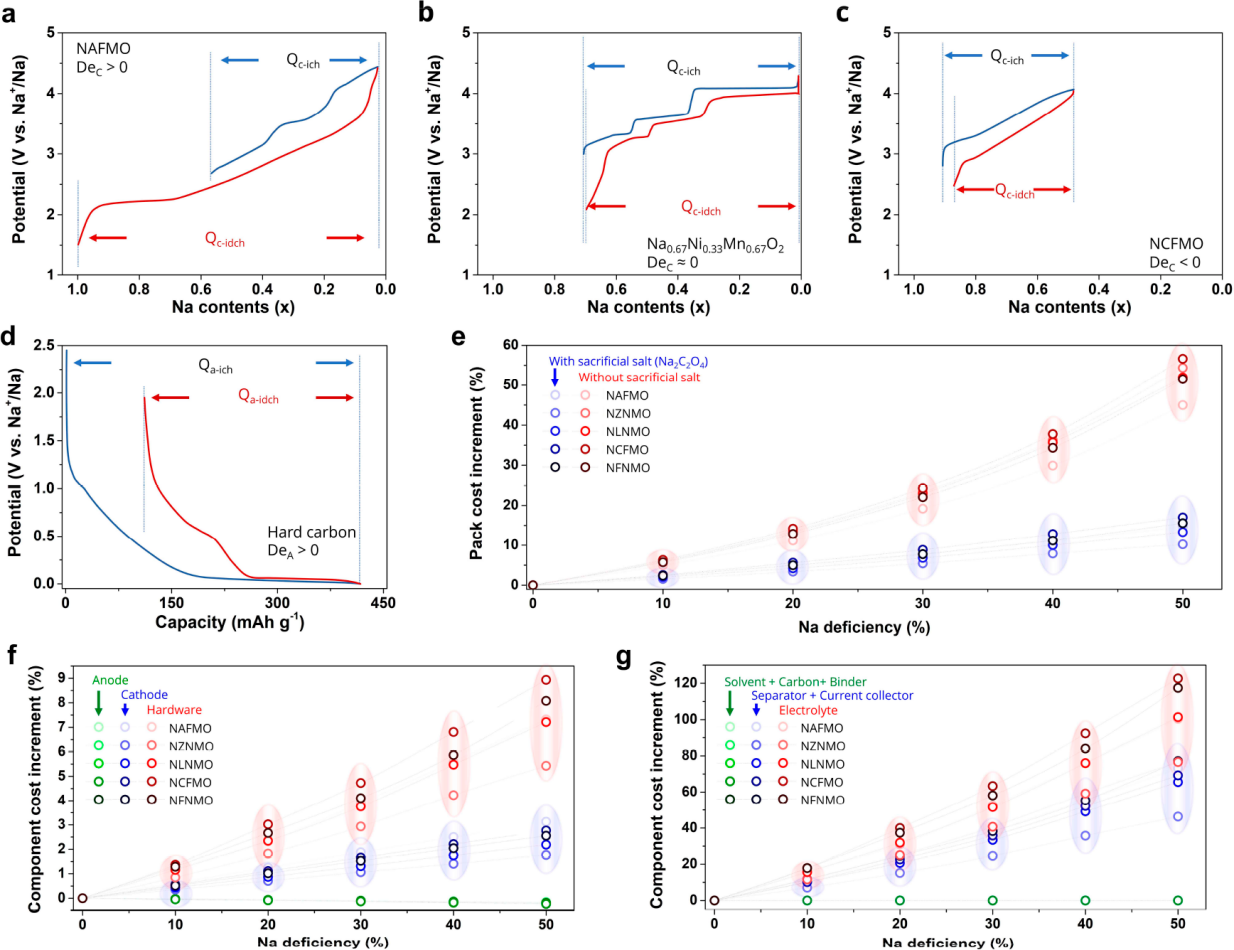
Na deficiency in Na_*x*_TMO_2_-based batteries. (a–c) Representative charge–discharge curves of (a) sodium-deficient, (b) sodium-sufficient, and (c) sodium-excess Na_*x*_TMO_2_ cathode materials. (d) Charge–discharge curves of hard carbon as the anode for NIBs. (e–g) Cost increments of (e) pack and (f, g) different components of various Na_*x*_TMO_2_-based NIBs with different extents of Na deficiency. Data were taken from refs (28), (47), (49), and (56).

Na cathodes can be classified into Na-deficient (Figure 7a), Na-sufficient (Figure 7b), and Na-excess (Figure 7c) types when their ICEs are higher, approximately equal to, and lower than 100%, respectively. Na deficiency is not closely related to Na content. For example, P2-type Na_0.67_Ni_0.33_Mn_0.67_O_2_ is a Na-sufficient cathode (Figure 7b), even though the initial Na content is lower than 1.0. In this case, Ni is the redox center, and according to its stoichiometry and the shift of its redox state between +2 and +4, no more than 0.67 stoichiometric units of Na can be reversibly inserted in the structure, as evidenced by the work of our group on the material.^28^ To simplify the discussion, we define the Na deficiency of the cathode (*D*_*e*,C_), anode (*D*_*e*,A_), and battery (*D*_*e*_) in eqs 2–4:
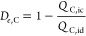
2
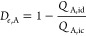
3
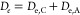
4where *Q*_C,ic_ (*Q*_C,id_) and *Q*_A,ic_ (*Q*_A,id_) are the initial charge (discharge) capacities of the cathode and anode, respectively.

The effects of Na deficiency (*D*_*e*_, eq 4) on the pack cost of grid-storage packs are evaluated. Without Na compensation strategies, the pack cost increases dramatically with the increment in *D*_*e*_ (red symbols in Figure 7e). For example, the pack cost of NZNMO-based NIBs increases from 6% to 23% to 54% when *D*_*e*_ is 10%, 30%, and 50%, respectively. Among the simulated NIBs, the NAFMO-based battery exhibits the lowest pack-cost increment due to its high energy density and low cost of cathode materials.

When a stoichiometric amount of sacrificial salt (Na_2_C_2_O_4_)^57^ is added in the cathode, the pack cost increment is decreased by 3–5 times compared to the case without sacrificial salt. As shown in Figure 7e (blue symbols), the pack-cost increment of NZNMO is 1.5%, 5%, and 10% for *D*_*e*_ of 10%, 30%, and 50%, respectively. When *D*_*e*_ is below 30%, the cost increments are similar for different NIBs, while at higher Na-deficient states, the cost increment is correlated to the working potential and energy density of the cathode material. For example, at 50% *D*_*e*_, the cost increments of NAFMO-, NZNMO-, NLNMO-, NCFMO-, and NFNMO-based NIBs are 15%, 10%, 13%, 17%, and 16%, respectively.

According to the cost-increment breakdown of NIB packs (Figure 7f,g), *D*_*e*_ exerts little influence on the cost of the anode and electrode-preparation compounds and only has a minimal effect on the cost of the cathode. At 50% *D*_*e*_, the highest cost increment of the evaluated Na cathodes is only 3.1%. However, the utilization of this sacrificial salt results in increased porosity and volume of the cathode due to the voids left by the decomposition of the salt itself. Therefore, this leads to a cost increment of hardware, separator plus current collectors, and electrolyte, all cost items related to the size of the battery. Nevertheless, the cost increase of the NIBs with sacrificial salt is still lower than the one without this compensation strategy.

## 4 Outlook and Conclusion

In this Account, we have reviewed the properties, challenges, and costs of Na_*x*_TMO_2_ cathodes and Na_*x*_TMO_2_-based full battery packs. In general, Fe-, Mn-, Cu-, and Ni-based Na_*x*_TMO_2_ materials are qualified cathodes for cost-effective NIBs. The cost gap between NIBs and LIBs has decreased dramatically due to the cost increase of raw materials for LIBs. In our opinion, Na_*x*_TMO_2_ based NIBs can be alternatives and/or complementary solutions to LIBs in large-scale implementations. Important challenges and perspectives for cost-effective NIBs are listed below.

(1) Expensive and scarce elements, in particular Li and Co, should be carefully considered before utilization. Although Li and Co improve the structural stability and reversible capacity of Na_*x*_TMO_2_, their supply risk and turbulent price substantially offset the cost advantage of NIBs. For example, the addition of 10 atom % Li in the active material, i.e., Na[Li_0.10_Ni_0.35_Mn_0.55_]O_2_, results in the highest cost among the simulated Na cathodes. This will be further increased when considering the battery scale.
(2) Developing high-voltage Na cathodes is essential for cost-effective NIBs. Mass cost, working potential, and specific/volumetric capacity might contribute equally to the cost of cathode materials. However, working potential ranks as the highest to further improve the competitiveness of NIBs. First, for a given required power density, a higher current density is necessary for the batteries with lower operational voltage. Second, the higher average working potential of the hard carbon anode makes it harder to fully utilize the capacity of cathodes in the low state-of-charge region.
(3) Three primary directions are highlighted to further optimize the cost of NIBs. The first is to pursue higher-energy-density cathodes, which not only decreases the mass of active material but also saves electrode-preparation compounds (binder, conductive additives, and solvent for making cathode slurries), current collectors, and electrolyte. Another critical direction is to reduce the need and cost of anode, binder, and electrolyte, which could be achieved respectively by lowering the unit cost and volume of electrolyte, transitioning from PVdF to bioderived water-soluble binders, and adopting advanced hard carbons with higher energy density, improved ICE, and cost-effective precursors. Third, adopting strategies to compensate for the Na deficiency in full cells and increasing the starting sodium content of cathodes will be important for the practical implementation of NIBs.

## Supporting Information Available

Additional analysis details, parameters, materials, and methods, including sources for cost and energy simulations (PDF)
