# Supplementary material for: Layered Oxide Cathodes for Sodium-Ion Batteries: Storage Mechanism, Electrochemistry, and Techno-economics
Source: Acc Chem Res. 2023 Jan 25;56(3):284–96. doi: 10.1021/acs.accounts.2c00690 (PMC9910041; doi:10.1021/acs.accounts.2c00690)
Supplement: Supplementary file 1 — ar2c00690_si_001.pdf [file ar2c00690_si_001.pdf]

Supporting information

Layered Oxide Cathodes for Sodium-Ion Batteries: Storage Mechanism, Electrochemistry, and Techno-Economics

Wenhua Zuo[a,b,†], Alessandro Innocenti[a,b,†], Maider Zarrabeitia[a,b,], Dominic Bresser[a,b,], Yong Yang[c,*], Stefano Passerini[a,b,d,*]

[a] *Helmholtz Institute Ulm (HIU), Helmholtzstrassse 11, D-89081 Ulm, Germany, E-mail: stefano.passerini@kit.edu*

[b] *Karlsruhe Institute of Technology, P.O. Box 3640, Karlsruhe 76021, Germany*

[c] *State Key Laboratory for Physical Chemistry of Solid Surfaces, and Department of Chemistry, College of Chemistry and Chemical Engineering, Xiamen University, Siming South Road 422, Xiamen 361005, People's Republic of China, Email: yyang@xmu.edu.cn*

[d] *Chemistry Department, Sapienza University, Piazzale A. Moro 5, 00185 Rome, Italy*

[†] *These authors contributed equally to this work*

General methods

The Battery Performance and Cost (BatPaC) 5.0 model[1] is utilized to compare the energy density and the cost of different NIBs and LIBs. The prices of raw materials in January 2020 and April 2022 are both utilized to evaluate the impact of price turbulence of raw materials on the pack cost of NIBs and LIBs with different battery sizes, *i.e.* a small domestic battery system of 7 kW and 11.5 kWh, a plug-in hybrid electric vehicle (pHEV) pack of 110 kW and 15 kWh, a high-end full electric vehicle (EV) pack of 150 kW and 100 kWh, and a grid storage pack of 250 kW and 500 kWh. Five most promising Na_xTMO_2 cathodes, P2-type $Na_{0.67}[Al_{0.1}Fe_{0.05}Mn_{0.85}]O_2$ (NAFMO),[2] P2-type $Na_{0.66}[Ni_{0.26}Zn_{0.06}Mn_{0.67}]O_2$ (NZNMO),[3,4] O3-type $Na[Li_{0.10}Ni_{0.35}Mn_{0.55}]O_2$ (NLNMO),[5] O3-type $Na_{0,9}[Cu_{0.22}Fe_{0.30}Mn_{0.48}]O_2$ (NCFMO),[6,7] and O3-type $Na[Fe_{0.40}Ni_{0.30}Mn_{0.30}]O_2$ (NFNMO),[8] and a biomass-derived hard carbon anode[9] have been chosen to simulate the cost of NIBs. NAFMO-based NIB is simulated in fully sodiation state of $Na[Al_{0.1}Fe_{0.05}Mn_{0.85}]O_2$ with stoichiometric amount of $Na_2C_2O_4$ as sacrificial salt.[10] As references, LIBs based on $LiNi_{0.5}Mn_{0.3}Co_{0.2}O_2$ (NMC 532, 4.3 V), $Li[Ni_{0.8}Co_{0.15}Al_{0.05}]O_2$ (NCA, 4.3 V), $LiMn_2O_4$ (LMO), and $LiFePO_4$ (LFP) cathodes coupled with graphite are selected. The representative charge-discharge profiles of Li cathodes from authors' labs and Na cathodes from references[2,4-6,8] are presented in Figure 4a and Figure 4b, respectively.

The detailed description of the BatPac model can be found in the report published by the Argonne National Lab.[1] Briefly, the model simulates battery packs of a determined rated power and capacity, taking into account the cost of the materials for the cells (active materials, conductive carbon, binder, separator, electrolyte, current collectors), the cost of the cell, module and pack hardware (casings, pack current collectors, cooling system), the cost of labor, the investment costs for the production site and other overheads. A yearly production of 500,000 battery packs is assumed here.

The density of the materials has been taken from the crystallographic values for lithium-ion cathodes, sodium-ion cathodes and graphite, and measured with a helium pycnometer for the hard carbon (1.55 g/cm^3).

The prices of the raw materials in January 2020 and April 2022 were obtained by comparing various sources (academic papers, databases for price of metals and chemical compounds, bulk chemical vendors), as seen in table S1, which can also be found in the Excel file of the Supplementary materials.

Table S1. the mass cost and energy cost of cathodes

**Material**	**Price, Jan 2020 [$/kg]**	**Price, Apr 2022 [$/kg]**	**Change [%]**	**Reference(s)**
Lithium carbonate	7.20	78.00	983%	https://tradingeconomics.com/commodity/lithium https://www.metal.com/Chemical-Compound/201102250059
Sodium carbonate	0.25	0.40	60%	https://tradingeconomics.com/commodity/soda-ash
Nickel sulfate hexahydrate	3.85	7.30	90%	https://www.metal.com/Nickel/201908270001 https://tradingeconomics.com/commodity/nickel
Cobalt sulfate heptahydrate	8.20	18.60	127%	https://www.metal.com/Chemical-Compound/201102250381 https://tradingeconomics.com/commodity/cobalt
Manganese sulfate monohydrate	0.85	1.70	100%	https://www.metal.com/Manganese/201805300001 https://tradingeconomics.com/commodity/manganese
Aluminum nitrate nonahydrate	0.45	0.80	78%	https://www.made-in-china.com/products-search/hot-china-products/Aluminum_Nitrate_Price.html https://tradingeconomics.com/commodity/aluminum
Zinc sulfate monohydrate	0.60	1.00	67%	https://tradingeconomics.com/commodity/zinc https://dir.indiamart.com/impcat/zinc-sulphate-monohydrate.html
Copper sulfate pentahydrate	1.96	3.00	53%	https://www.chemanalyst.com/Pricing-data/copper-sulphate-1163 https://tradingeconomics.com/commodity/copper
Iron sulfate heptahydrate	0.10	0.15	50%	https://www.made-in-china.com/price/iron-sulfate-price.html https://tradingeconomics.com/commodity/ironore62
Manganese dioxide	1.50	3.12	108%	https://www.metal.com/Manganese/202105130001 https://tradingeconomics.com/commodity/manganese
Iron phosphate	2.67	4.00	50%	https://tradingeconomics.com/commodity/ironore62 https://www.metal.com/Ternary-precursor-material/202111010002
Glucose	0.70	0.60	-14%	https://www.selinawamucii.com/insights/prices/united-states-of-america/glucose/

				https://www.alibaba.com/showroom/glucose-price-per-ton.html
Sodium oxalate	1.00	1.60	60%	https://dir.indiamart.com/impcat/sodium-oxalate.html

Material	Price, Jan 2020 [$/m²]	Price, Apr 2022 [$/m²]	Change [%]	Reference(s)
Copper foil (8 μm)	0.90	1.13	26%	https://tradingeconomics.com/commodity/copper; https://www.metal.com/Copper
Aluminum foil (15 μm)	0.20	0.25	25%	https://tradingeconomics.com/commodity/aluminum; https://www.metal.com/Aluminum
Separator (15 μm)	0.16	0.14	-13%	https://www.metal.com/Ternary-precursor-material

Material	Price, Jan 2020 [$/l]	Price, Apr 2022 [$/l]	Change [%]	Reference(s)
Organic electrolyte	12.9	24.7	91%	https://www.metal.com/Ternary-precursor-material

Material	Formula	Price, Jan 2020 [$ kg^{-1}]	Price, Apr 2022 [$ kg^{-1}]	Price, Jan 2020 [$ kWh^{-1}]	Price, Apr 2022 [$ kwh^{-1}]
NMC 532	$Li[Ni_{0.5}Mn_{0.3}Co_{0.2}]O_2$	21.71	60.19	30.84	85.52
NCA	$Li[Ni_{0.8}Co_{0.15}Al_{0.05}]O_2$	23.37	62.97	30.11	81.12
LMO	$Li[Mn_2]O_4$	11.16	28.55	23.43	59.94
LFP	$LiFePO_4$	12.46	30.31	24.21	58.91
NAFMO (w/ SS)	$Na[Al_{0.1}Fe_{0.05}Mn_{0.85}]O_2$	10.22	11.96	25.34	29.67
NZNMO	$Na_{0.66}[Ni_{0.26}Zn_{0.07}Mn_{0.67}]O_2$	12.08	15.59	31.85	41.11
NLNMO	$Na[Li_{0.1}Ni_{0.35}Mn_{0.55}]O_2$	12.48	20.18	33.76	54.56
NCFMO	$Na_{0.9}[Cu_{0.22}Fe_{0.30}Mn_{0.48}]O_2$	10.36	11.85	34.76	39.77
NFNMO	$Na[Fe_{0.4}Ni_{0.3}Mn_{0.3}]O_2$	11.80	14.93	31.95	40.42

The analysis took into account also the change in prices of the organic electrolyte, the separator and the aluminum and copper current collectors. As conservative hypothesis, the price of the organic electrolyte for LIBs and SIBs has been considered equal, both in January 2020 and April 2022. As a matter of fact, the substitution of

$LiPF_6$ with $NaPF_6$ in the organic electrolyte is assumed to not significantly change the cost of the electrolyte, as evidenced by other works, since the cost of the cation in the salt is just a minimal fraction of the cost of the salt itself.[11-13]

The following assumptions were made in the modeling of the production processes:

- The modeled reactions are 1) for multi-transition metal layered oxides, the co-precipitation of the transition metal sources with sodium carbonate in water in a stirred reactor and the calcination of the precipitate mixture with sodium/lithium carbonate, and 2) for LFP and LMO, a solid state reaction, i.e., the calcination of the milled precursors mixture.
- The transition metal sources for the production of layered oxides are the hydrated sulfate salts, except for aluminum, for which the hydrated nitrate salt was employed. The LFP uses lithium carbonate, iron phosphate and glucose (for the carbon coating) as precursors, while LMO has lithium carbonate and electrolytic manganese dioxide.
- A fixed cost of 17 M$/year has been assumed for every production process, which is added to the costs of the raw materials to obtain the final cost of the cathode. With an assumed output of the plant of 6500 kg/day and 320 days of operation per year, this results in an additional 8 $/kg in the final cost of the cathode other than the raw material costs. This figure was estimated after the comparison of different references about the quantification of fixed and operative costs for cathode production plants of sizes similar to the one modeled in this work.

The price of the graphite (for LIBs) and hard carbon (for SIBs) anodes have been set respectively to 12.50 $/kg (the standard BatPac 5.0 value) and 10$/kg for both January 2020 and April 2022.

The characteristics of the four types of battery packs considered in the simulations are summarized in table S2.[1]

Table S2 - Characteristics of the simulated battery packs

Parameter	Domestic storage battery	Grid storage battery	pHEV battery	EV battery
Number of cells per module (total)	36	4	20	20
Number of cells in parallel group in module	1	1	1	4
Number of modules in row	2	42	2	5
Number of rows of modules per pack	1	7	1	4
Number of modules in parallel	1	7	1	1
Number of packs manufactured per year	500,000	500,000	500,000	500,000
Pack energy, kWh	11.5	500	15	100
Target battery pack power at 20% SOC, kW	7	250	110	150
Power-to-energy ratio	0.61	0.5	7.3	1.50

The effect of the cycle life of the different types of cathodes can be seen looking at tables S3 and S4, where we make an example by assigning a certain cycle life QR (defined as the number of cycles n after which the capacity retention of the battery reaches 80%) to selected cathode materials. The related coulombic efficiency CE is calculated through the formula:

$$\mathrm{CE}\ [\%] = \sqrt[n]{\mathrm{QR}} \cdot 100$$

Steps of 1000 cycles up to 5000 cycles are considered, and if the cycle life is reached within the step, the cost of the battery pack is added again, as if a new battery of the same type has to be purchased to substitute the one that reached its end-of-life. This simplified analysis of the cost of ownership can illustrate which impact has the coulombic efficiency of the battery on its total cost.

Since it is not appropriate to compare the cycle life of commercial and pouch cylindrical cells (as in the case of lithium-ion cathodes, where there is abundancy of data) and of laboratory-scale cells (as in the case of layered oxide sodium-ion cathodes, which are

still in the development stage and no studies have been published on commercial-scale cells, except for one on a sodium-vanadium phosphate cathode), we assume three cases for a selected sodium-ion cathode, NZNMO: poor cyclability, with a cycle life of 200 cycles; good cyclability, with a cycle life of 500 cycles; excellent cyclability, with a cycle life of 1000 cycles.

The lower the cycle life (as in the case of LMO and NZNMO with the poor cyclability), the higher is the cost in time and it is strongly affected by the initial cost of the battery. The NZNMO on the long run can be compared with NMC or NCA only if the cycle life is near 1000 cycles for the January 2020 case, but in the April 2022 case, due to the higher initial cost of the lithium-ion batteries, the cost of ownership is the same nearer to 500 cycles of cycle life for the NZNMO.

Table S3 - Effect of cycle life on the cost of ownership of the domestic energy storage battery pack for the lithium-ion batteries and NZNMO (January 2020 costs)

Battery	Cycle life [cycles]	Coulombic efficiency	Cost of ownership at different cycle number [$/kWh]					
			Cycle n°0	Cycle n°1000	Cycle n°2000	Cycle n°3000	Cycle n°4000	Cycle n°5000
**NMC 532**	500	99.95538%	170	509	849	1189	1528	1868
**NCA**	500	99.95538%	170	511	852	1193	1534	1875
**LFP**	3000	99.99256%	171	171	171	341	341	341
**LMO**	300	99.92564%	167	668	1169	1837	2338	2839
**NZNMO (poor cyclability)**	200	99.88849%	245	1470	2695	3919	5144	6369
**NZNMO (good cyclability)**	500	99.95538%	245	735	1225	1715	2205	2695
**NZNMO (excellent cyclability)**	1000	99.97768%	245	490	735	980	1225	1470

Table S4 - Effect of cycle life on the cost of ownership of the domestic energy storage battery pack for the lithium-ion batteries and NZNMO (April 2022 costs)

Battery	Cycle life [cycles]	Coulombic efficiency	Cost of ownership at different cycle number [$/kWh] Cycle n°0	Cycle n°1000	Cycle n°2000	Cycle n°3000	Cycle n°4000	Cycle n°5000
**NMC 532**	500	99.95538%	247	740	1233	1726	2219	2712
**NCA**	500	99.95538%	245	736	1227	1718	2208	2699
**LFP**	3000	99.99256%	221	221	221	443	443	443
**LMO**	300	99.92564%	222	887	1551	2438	3103	3768
**NZNMO (poor cyclability)**	200	99.88849%	278	1667	3056	4446	5835	7224
**NZNMO (good cyclability)**	500	99.95538%	278	834	1389	1945	2501	3056
**NZNMO (exc. cyclability)**	1000	99.97768%	278	556	834	1111	1389	1667

References

(1) Nelson, P. A.; Ahmed, S.; Gallagher, K. G.; Dees, D. W.: Modeling the performance and cost of lithium-ion batteries for electric-drive vehicles. Third Edition ed.; Argonne National Lab. (ANL), Argonne, IL: Argonne National Lab. (ANL), Argonne, IL, 2019; https://www.osti.gov/biblio/1209682.

(2) Liu, X.; Zhong, G.; Xiao, Z.; Zheng, B.; Zuo, W.; Zhou, K.; Liu, H.; Liang, Z.; Xiang, Y.; Chen, Z.; Ortiz, G. F.; Fu, R.; Yang, Y. Al and Fe-containing Mn-based layered cathode with controlled vacancies for high-rate sodium ion batteries. *Nano Energy* **2020**, *76*, 104997.

(3) Wu, X.; Guo, J.; Wang, D.; Zhong, G.; McDonald, M. J.; Yang, Y. P2-type $Na_{0.66}Ni_{0.33-x}Zn_xMn_{0.67}O_2$ as new high-voltage cathode materials for sodium-ion batteries. *J. Power Sources* **2015**, *281*, 18-26.

(4) Zuo, W.; Qiu, J.; Hong, C.; Liu, X.; Li, J.; Ortiz, G. F.; Li, Q.; Zheng, S.; Zheng, G.; Yang, Y. Structure-performance relationship of Zn^{2+} substitution in P2-$Na_{0.66}Ni_{0.33}Mn_{0.67}O_2$ with different Ni/Mn ratios for high energy sodium-ion Batteries. *ACS Appl. Energy Mater.* **2019**, *2*, 4914-1924.

(5) Zheng, S.; Zhong, G.; McDonald, M. J.; Gong, Z.; Liu, R.; Wen, W.; Yang, C.; Yang, Y. Exploring the working mechanism of Li^+ in O3-type $NaLi_{0.1}Ni_{0.35}Mn_{0.55}O_2$

cathode materials for rechargeable Na-ion batteries. *J. Mater. Chem. A* **2016**, *4*, 9054-9062.

(6) Mu, L.; Xu, S.; Li, Y.; Hu, Y. S.; Li, H.; Chen, L.; Huang, X. Prototype sodium-ion batteries using an air-stable and Co/Ni-free O3-layered metal oxide cathode. *Adv. Mater.* **2015**, *27*, 6928-6933.

(7) Liu, Q.; Lin, W.; Liang, C.; Guo, Y. Positive electrode active material and its preparation method, sodium ion battery and apparatus containing the sodium ion battery. United States Patent.

(8) Kuze, S.; Kageura, J.-i.; Matsumoto, S.; Nakayama, T.; Makidera, M.; Saka, M.; Yamaguchi, T.; Yamamoto, T.; Nakane, K. Development of a sodium ion secondary battery. *Sumitomo Kagaku* **2013**, *2013*, 1.

(9) Moon, H.; Zarrabeitia, M.; Frank, E.; Böse, O.; Enterría, M.; Saurel, D.; Hasa, I.; Passerini, S. Assessing the Reactivity of Hard Carbon Anodes: Linking Material Properties with Electrochemical Response Upon Sodium- and Lithium-Ion Storage. *Batteries & Supercaps* **2021**, *4*, 960-977.

(10) Niu, Y. B.; Guo, Y. J.; Yin, Y. X.; Zhang, S. Y.; Wang, T.; Wang, P.; Xin, S.; Guo, Y. G. High-Efficiency Cathode Sodium Compensation for Sodium-Ion Batteries. *Adv. Mater.* **2020**, *32*, e2001419.

(11) Vaalma, C.; Buchholz, D.; Weil, M.; Passerini, S. A cost and resource analysis of sodium-ion batteries. *Nat. Rev. Mater.* **2018**, *3*, 18013.

(12) Roberts, S.; Kendrick, E. The re-emergence of sodium ion batteries: testing, processing, and manufacturability. *Nanotechnol Sci Appl* **2018**, *11*, 23-33.

(13) Greim, P.; Solomon, A. A.; Breyer, C. Assessment of lithium criticality in the global energy transition and addressing policy gaps in transportation. *Nat. Commun.* **2020**, *11*, 4570.
